# 

**Published:** 1853-01

**Authors:** 


### PROF. HARRIS' PRINCIPLES AND PRACTICE OF DENTAL SURGERY.

The fifth edition of this standard work is just from the press of the enterprising publishers, Lindsley & Blackiston, Phil. It is gotten up in their usual style of elegance. This work has grownf rom a medium sized volume (the first edition), to a work of some eight hundred pages. The work is divided for con venience and systematic order into seven parts, commencing with the anatomy and physiology of the mouth, tracing out the development of the dental org[illegible]s, &c.; next the physical characteristics of the teeth, gums, &c.; third the diseases of the teeth, treatment, &c.; fourth, diseases of the gums, alveolar processes, &c.; fifth, diseases of the maxillary sinus and treatment; sixth, mechanical dentistry; and seventh, diseases and defects of the palatine organs. The work throughout is illustrated with a large number of very excellent engravings. The continued demand for this work is perhaps its best eulogium; and the Dr. has in each addition aimed to keep up with the improvements in the profession; hence that part appropriated to mechanical dentistry is much enlarged, and embraces the recent improvements introduced by Drs. Hunter and Allen.

As a practical work adapted to the wants of the dental student, it is entirely the best work extant.

It is for sale by Dr. J. M. Brown, Dental Depot, Cincinnati, Ohio. Also, at the principle book stores in the city.

The *Southern Journal* of the *Medical* and *Physical Sciences* devoted to medicine, surgery, chemistry, pharmacy, dental science, reviews, &c. We have received the prospectus of the above work; but not as yet the first number of the work itself. It is to be edited by John W. King, M. D; W. P. Jones, M. D.; Richard O. Carrey, M. D.; and B. Wood, M. D., Dentist. It is to be published bimonthly, making at the end of the year, a volume of 432 pages at $2 per annum *always in advance.* We feel assured that Dr. Wood will well sustain the dental department.

The first number of the above work is just received.

### NEW DENTAL WORKS.

The editors of the American Journal of Dental Science notice the following works as in preparation for the press:

1st. History of American Dentistry, Biography, &c.; by Dr. W. H. Dwinelle. There is material here for a rich and interesting volume, and we have no doubt the Dr. will do it justice. 2nd. A work by the same on microscopical anatomy. We shall be rejoiced to see such a work from an American author. 3d. A Treatise on Dental Chemistry and Metallurgy, by Professor A. L. Piggot, M. D., Baltimore. A well written work on this subject is now very much needed, and we hope the Dr. will give us one which will be suitable for a text book in our Dental Colleges.

### DENTAL EXPOSITOR.

The last number of this work is received, and contains Dr. Brown's admirable poem *Dental Hygeia.* We feel assured that the forthcoming numbers will be looked for with much interest by the Profession. They are to contain the Drs. Essays in Mechanical Dentistry, revised and brought up to the present improved condition of dental science. They cannot fail to be useful to the practical DENTIST.

### MISSISSIPPI VALLEY ASSOCIATION OF DENTAL SURGEONS.

The members of this Association will please bear it in mind that the time for their regular meeting has been changed from September to February; and that the meeting takes place on the 17th and 18th of Feb. next, in the Ohio College of Dental Surgery.

### RECEIPTS FOR VOL. 5 DENTAL REGISTER.

Dan'l. Dougherty, Bardstown, Ky.
Joseph Sanfond, Chillicothe, O.
W. B. Ross, Newport, Ky.
T. B. Hamlin, Nashville, Tenn.
W. R. Scott, Raleigh, N. C.
H. N. Ferm, Rochester, Ky.
A. S. Daryer, Greenfield, O.
S. D. Muse, Sharon, Miss.
W. E. Ide, Columbus, O.
W. H. Goddard, Louisville, Ky.
M. D. Stoneman, Westfield, Ia.
Nimrod Hutt, Hillsboro', O,
D. W. Harris, West Liberty, O.
J. A. Cleaveland, Charleston, S. C.
N. Perrin, Georgetown, Ky.
N. P. Allen, Smith Grove, Ky.
S. L. Mentzer, Phila. Pa., for vols. 1, ,2 3, 4, and 5.
Dr. Meredith, Cin., vols. 4 and 5.

### RECEIPTS FOR VOL. 6, DENTAL REGISTER.

W. C. Duncan, Cin. O.
B. A. Satterthwaite, Lima, O.
Dan'l. Dougherty, Bardstown, Ky.
Joseph Sanford, Chillicothe, O., on vol. 7 $1.
J. Scott, Pittsburgh, Pa.
John Harris, Salem, O.
E. S. Holmes Lockport, N. Y.
J. A. Reeves, Mt. Vernon, O.
T. R. Willard, Dayton, O.
J. C. Whinery, Salem, O.
John W. Keely, Brookville, Ia.
Wm. Bell, Elizaville, Ky.
J. B. Beauman, Mt. Vernon, O.
W. R. Jeffries, Indianalaisp, Ia.
W. G. Redman, Henderson, Ky.

### PROSPECTUS

OF THE

### DENTAL REGISTER OF THE WEST.

The Register has now completed its fifth volume, and it will be seen by a resolution of the Mississippi Valley Association, that the work has been given into our hands for future management.

The question has been now practically solved that such a work can be sustained by the Profession, and we presume that no one will doubt but that a work of the kind is needed.

Believing that the interest of the Profession in the West, demand a channel for the dissemination of useful knowledge. We have determined to continue the publication of the Register, and will now say to the Profession that it will be made as valuablein reading matter, and as neat in mechanical execution, as their liberality will possibly justify. There are two things absolutely essential to make it such a work as we should like to see in the hands of the Profession. The first is money to "pay the printer" and the other (which is equally important), well written articles for its pages. The former we are satisfied, is in the hands of the Profession, and if they deliberately view the condition of Dental Science, the progress that is being made, and the need of periodical publications, to keep pace with the improvements of the age, and to collect and preserve such for future use, will be cheerfully furnished. If the Profession East, with the aid of the West, sustain four such publications? should not the West with the aid of the East sustain one?

In general enterprise—in ambition to excel, and all the elements for the advance of Dental Science—we are not behind as we believe any portion of the world, and should not this be developed, and made to tell on this land of future renown? We think it should.

The latter, which is all important, and without which, all the gold in California would be of no avail, we believe, if not in the hands, is in the *heads* of the Profession. There is talent, which if properly exercised would do much to elevate Dental Science. Many of our practitioners are doing but little to make known the result of their experience. They should bear in mind that what to them appears old and common, may be new and of interest to others.

The columns of the Register shall always be open to the profession, for the publication of well written practical or theoretical matter which may be of general interest. We shall, however, aim to keep its columns clear of all which is of a party, personal, or abusive character.

The size of the Register, style of execution, &c. will be much the same as the last volume, and will be published quarterly, at $2 per annum; to those paying in advance, postage free. JAMES TAYLOR.

